# Estimating the impact of public health strategies on the spread of SARS‐CoV‐2: Epidemiological modelling for Toulouse, France

**DOI:** 10.1002/rmv.2224

**Published:** 2021-03-13

**Authors:** Chloé Dimeglio, Marcel Miedougé, Jean‐Michel Loubes, Jean‐Michel Mansuy, Jacques Izopet

**Affiliations:** ^1^ UMR Inserm U1043 UMR CNRS U5282 Centre de Physiopathologie de Toulouse Purpan (CPTP) Toulouse France; ^2^ Virology Laboratory Centre Hospitalier Universitaire (CHU) de Toulouse Hôpital Purpan Toulouse France; ^3^ Toulouse Mathematics Institute Université de Toulouse Toulouse France

**Keywords:** public health, SARS‐CoV‐2, statistical model

## Abstract

The spread of severe acute respiratory syndrome coronavirus 2 (SARS‐CoV‐2) and the resulting disease COVID‐19 has killed over 2 million people as of 22 January 2021. We have used a modified susceptible, infected, recovered epidemiological model to predict how the spread of the virus in France will vary depending on the public health strategies adopted, including anti‐COVID‐19 vaccination. Our prediction model indicates that the French authorities' adoption of a gradual release from lockdown could lead in March 2021 to a virus prevalence similar to that before lockdown. However, a massive vaccination campaign initiated in January 2021 and the continuation of public health measures over several months could curb the spread of virus and thus relieve the load on hospitals.

**Abbreviations**
MERS‐CoV: Middle East respiratory syndrome coronavirus
SARS‐CoV‐2: severe acute respiratory syndrome coronavirus 2
SIR: susceptible, infectious and recovered
WHO: World Health Organization; MERS‐CoV, Middle East respiratory syndrome coronavirus

## 1 INTRODUCTION

The severe acute respiratory syndrome coronavirus 2 (SARS‐CoV‐2), which emerged in Wuhan, China, in December 2019, spreads mainly by sustained human‐to‐human transmission.^1^ This spread was so rapid that the World Health Organization (WHO) declared the resulting disease a pandemic.^2^ Many countries opted for a strict lockdown in March 2020 to slow the epidemic and protect their health services. But SARS‐CoV‐2 resumed its rampage in Europe, including France, at the end of the summer. The measures taken by several large cities to limit virus transmission have provided data that can be used to quantify the impact of measures such as mask wearing, restricting access to public spaces and curfew, on its proliferation.^3^ The French authorities decided to establish a new lockdown from 29 October 2020 to 28 November 2020, followed by a gradual release from lockdown. Two more steps of lockdown release are planned for 15 December 2020 and 20 January 2021. This is likely to coincide with the approval of first‐generation COVID‐19 vaccines at the end of 2020 or early 2021.^4^ These vaccines could provide several benefits, protecting individuals from COVID‐19 symptoms and stimulating population immunity resulting in reduced SARS‐CoV‐2 transmission. However, the impact of these anti‐COVID‐19 vaccines on infection and thus transmission has not yet been assessed.

We examined the impact of different public health strategies on the spread of SARS‐CoV‐2 using data for the French city of Toulouse. We focused on the impact of the different end‐of‐lockdown strategies on the resurgence of the epidemic, based on the strategy adopted by France. We also evaluated the potential impact of the vaccination campaign that was launched on 1 January 2021 on the spread of the virus.

## 2 METHODS

### 2.1 Statistical model

Earlier models for SARS‐CoV‐2 were based on published positive cases and do not take into account the patients' ages or any evolutive diffusion coefficient.^5^, ^6^ That is probably why the Johns Hopkins University predictive model underestimated the spread of the virus in Italy and overestimated its spread in France and the United Kingdom. Our model is a discretized version of a susceptible, infectious and recovered (SIR)‐type model.^7^ These compartmental models are well suited to studies of the spread of SARS‐CoV‐2 in different populations.^8^, ^9^ Our model includes a diffusion/transmission coefficient $R_{0}$ that varies with the likelihood of contagion, and two reduction coefficients $\hat{c}$and $\hat{q}$ that describe the impact of public health measures on virus transmission. The model predicts how the SARS‐CoV‐2 virus would have evolved and projects the daily percentage of new positive cases. By cumulative effect, we therefore obtain a projection of the seroprevalence of SARS‐CoV‐2 in France.

We have used four variables $\left(S_{n},\;P_{n},\;Q_{n},\;I_{n}\right)$, where $S_{n}$ is the number of healthy people on day n, and $P_{n}^{i}$ is the number of undetected contagious carriers infected for i days $\left(1\leq i\leq N_{T}\right)$. Similarly, $Q_{n}^{i}$ is the number of detected contagious carriers infected for i days $\left(1\leq i\leq N_{T}\right)$ on day n, and $I_{n}$ is the number of people who were immunized. We assume that the risk of reinfection by SARS‐CoV‐2 after a first infection is negligible.

$N_{T}$ is the number of days a person is contagious, and *α* is the percentage of the population tested each day. $R_{0}$ is the number of healthy people who a contagious person contacts and infects. We assume that $R_{0}$ varies over time and peaks when the virus load is maximal: 7 days after the start of infection.^10^, ^11^ We assume that the number of days a person is contagious is equal to the times of infection, that is, 20 days.^10^, ^12^ For all $1\leq i\leq N_{T}$, $R_{0}^{i}$ is

$$R_{0}^{i}=R_{0}.e^{-\frac{1}{2}{\left(\frac{i-7}{\sqrt{20}}\right)}^{2}}$$

N is the total population at the start of the epidemic phase, $\hat{c}$ is the multiplier for the pace of the epidemic throughout public health restriction phases $\left(0\leq \hat{c}\leq 1\right)$ and $\hat{q}$ is the same multiplier during the quarantine period $\left(0\leq \hat{q}\leq 1\right)$. $\hat{c}$ and $\hat{q}$ are set at 1 when there is no restriction or quarantine. The lower the values of$\;\hat{c}$ or $\hat{q}$, the greater the constraint which is applied to halt the spread of the virus. Some values of $\hat{c}$ and the value of $\hat{q}$ have been estimated in previous works by correcting the values predicted by the model using real data collected by the Toulouse Virology Laboratory.^3^, ^13^

∀n∈⟦d+1,+∞⟧ $\hat{c}$ is defined as $\hat{c}=\underset{c}{\text{argmin}}{\left|{\hat{P}}_{n}-P_{n}\left(c\right)\right|}_{n\;\in \;⟦1,\;d}⟧$

And $\forall \;n\;\in ⟦\;d^{\prime }+1,\;+\infty ⟧$ $\hat{q}$ is defined as $\;:\;\hat{q}=\underset{q}{\text{argmin}}{\left|{\hat{P}}_{n}-P_{n}\left(q\right)\right|}_{n\;\in \;⟦1,\;d^{\prime }}⟧$

We used data collected by the Toulouse Virology Laboratory from March 2020 to June 2020 to set $\hat{q}$ to 0.05.^13^ The values of $\hat{c}$ varied according to the public health restriction measures implemented in the Toulouse area.^3^

Nis given by

$$N=\;S_{n}+\;P_{n}+\;Q_{n}+\;I_{n}$$

On transition from day n to day n+1, we have

(1) $$\forall 1\leq i\leq N_{T}-1,P_{n+1}^{i+1}=P_{n}^{i}\;\left(1-\alpha \right)$$

According to Equation (1), the number of undetected contagious carriers on day n+1 is the number of untested, undetected carriers who were infected but not detected on day n.

(2) $$P_{n+1}^{1}=\frac{S_{n}}{N}.\left[\sum_{i}P_{n}^{i}.R_{0}^{i}.\hat{c}+\sum_{i}Q_{n}^{i}.R_{0}^{i}.\hat{q}\;\right]$$

According to Equation (2), the number of new undetected contagious carriers on day n+1 is the number of healthy people who were infected by undetected carriers at any stage of infection or by detected carriers at any stage of infection on day n.

(3) $$I_{n+1}=I_{n}+P_{n}^{N_{T}}+Q_{n}^{N_{T}}$$

According to Equation (3), the number of immunized people on dayn+1 corresponds to the number of people immunized on day n plus the people who were on their last day of infection on day n, whether or not they were tested.

$Q_{n+1}^{1}=0\;$(no quarantine on day one, test results needed)

(4) $$\forall \;1\leq i\leq N_{T}-1,\;Q_{n+1}^{i+1}=\;Q_{n}^{i}+P_{n}^{i}\;.\alpha$$

According to Equation (4), the number of detected contagious carriers on day n + 1 is defined as the number of detected contagious carriers on day n plus the number of tested, but undetected contagious carriers on day n.

$\text{We}\;\text{set}\;R_{0}=2.2$ at its peak, based on a national and regional French study,^14^ and the international evaluations of the WHO.^15^

### 2.2 Study population

We estimated the initial model settings using data collected by the Toulouse Virology Laboratory (Table 1). The total number of tests each day was 2500. The population of greater Toulouse is around 1 million (source: INSEE). We assumed that 3.2% of this population had been infected with SARS‐CoV‐2 at the end of the first lockdown (11 May 2020),^16^ which implies that there were close to 32,000 immune individuals, $I_{0},$ in mid‐May. The number of SARS‐CoV‐2 cases gradually increased from 21 July 2020 (time = 0 for the model). The date d corresponds to 5 November 2020.

**TABLE 1:** Model initial parameters

Age group	% in Toulouse population (Source: INSEE)	*∝* (based on Toulouse data)	*N*_*T*_	$\hat{C}$ **(Estimated by the model)**	$\hat{q}$ **(Estimated by the model)**
<15 years	14.8%	10.2%	20	cf Results section	0.05
15–60 years	68.2%	61.58%	20	cf Results section	0.05
60–74 years	10.4%	10.68%	20	cf Results section	0.05
m>75 years	5.5%	17.54%	20	cf Results section	0.05

The percentage of new cases of SARS‐CoV‐2 per day was predicted using the initial parameters (Table 1). This estimation was based on the number of cases on the previous day and a contagion parameter $(R_{0}^{i})\;$that varied according to the day of infection, and on the administration parameters (quarantine, lockdown or restriction phases). We assumed that the COVID‐19 vaccination campaign began on 1 January 2021. The theoretical efficacy of vaccination was set at 94% as stated by the Pfizer trial,^17^ and we consider a subject to be immunized 7 days after the second dose (i.e., 28 days after the first injection).

## 3 RESULTS

The match between the values predicted by the model and the values observed from 6 November 2020 to 1 January 2021 is given by *R* ^2^ = 86.1%.

### 3.1 End‐of‐lockdown strategies

**FIGURE 1:**
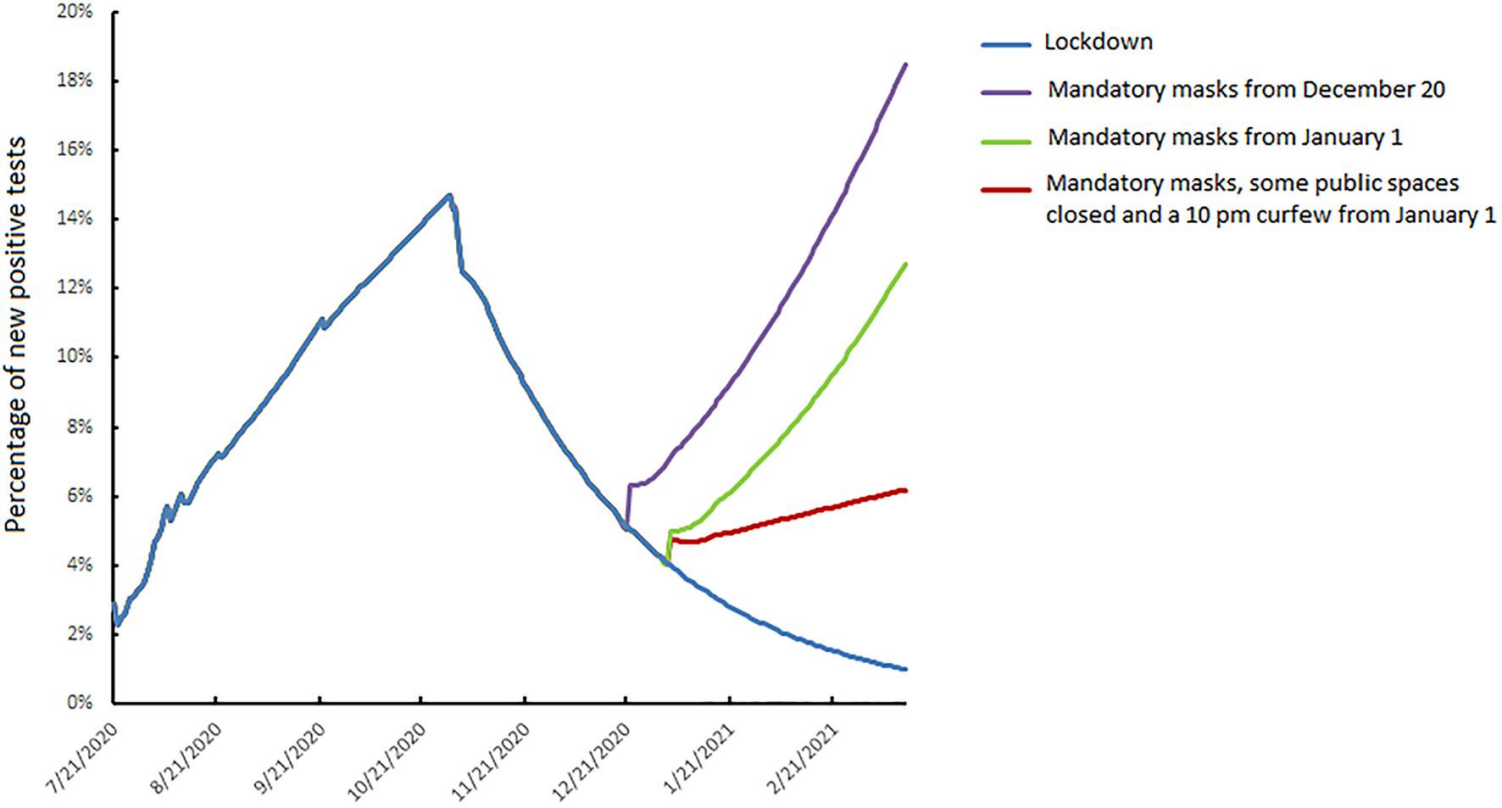
Daily dynamics of SARS‐CoV‐2 infection, 21 July 2020 to 14 March 2021: influence of lockdown strategies

The percentage of new positive cases peaked at around 16% of the population on 29 October 2020 (Figure 1). Lockdown interrupted the increase in SARS‐CoV‐2 positive cases by increasing the stress coefficient ($\hat{c}=$53%) (blue curve, Figure 1) so that only 7.5% of the people tested positive on 1 December 2020. We first looked at the result of an exit from lockdown followed by mandatory mask wearing alone when the estimated value of $\hat{c}$ became 72%. The virus spread would be difficult to control, whether lockdown ends on December 20 (purple curve), or January 2 (green curve). The model predicts that 10% of people would test positive on January 27 (December 20 end) or on February 24 (January 27 end). All these exit strategies would require a new lockdown. We then analysed the effect of a release from lockdown delayed to January 2021 accompanied by a return to the measures previously in force: compulsory mask wearing, closure of public spaces and a 10 pm curfew (red curve). This resulted in a $\hat{c}$ value of 63% and the 10% testing positive value would be reached only on April 28.

**FIGURE 2:**
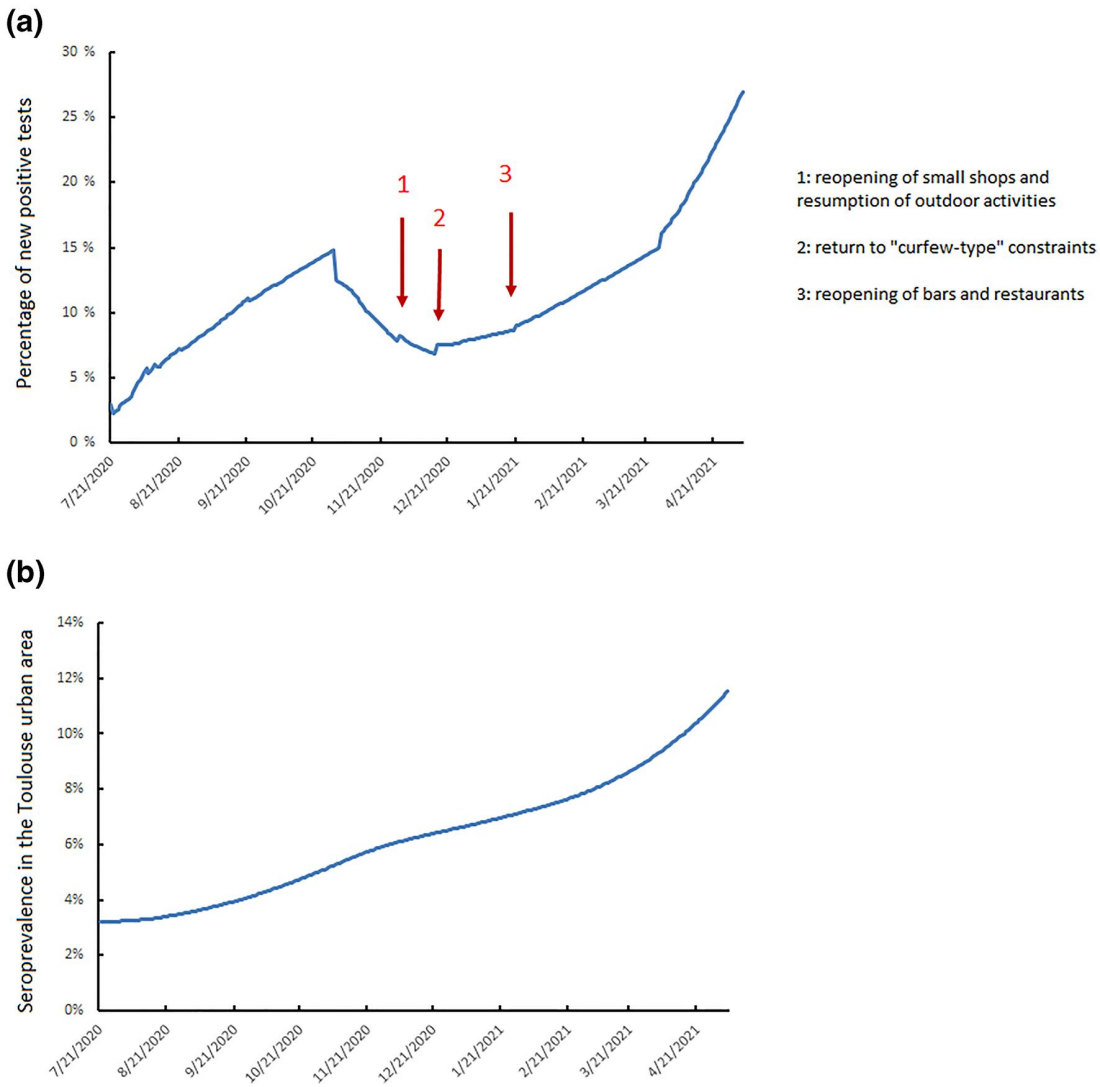
(a) Daily spread of SARS‐CoV‐2 infection, 21 July 2020 to 5 May 2021 depending on the containment strategy. (b) Cumulative SARS‐CoV‐2 seroprevalence in the Toulouse urban area from 21 July 2020 to 5 May 2021

Finally, the French authorities announced a release from lockdown in three stages (Figure 2a) with a first release from lockdown on November 28 (point 1, Figure 2a), reopening of small shops and the resumption of outdoor activities. The second lockdown was due to end on December 15 with a return to an 8 pm curfew (point 2, Figure 2a). Lastly, bars and restaurants will reopen on 20 January 2021 (point 3, Figure 2a), and universities 15 days later provided the virus spread has stabilized. Under these conditions, the percentage of SARS‐CoV‐2 positive tests could increase from the 7.5% who tested positive on December 1 to 10% at the beginning of February 2021 and reach 15% positive tests, similar to that before the second lockdown, one month later (March 2021) (Figure 2a).

### 3.2 Consequences of SARS‐CoV‐2 vaccination

As the SARS‐CoV‐2 epidemic will resume, despite any or all the measures adopted by the French authorities, mass vaccination must be rapidly introduced to limit virus spread and control the COVID‐19 epidemic.

**FIGURE 3:**
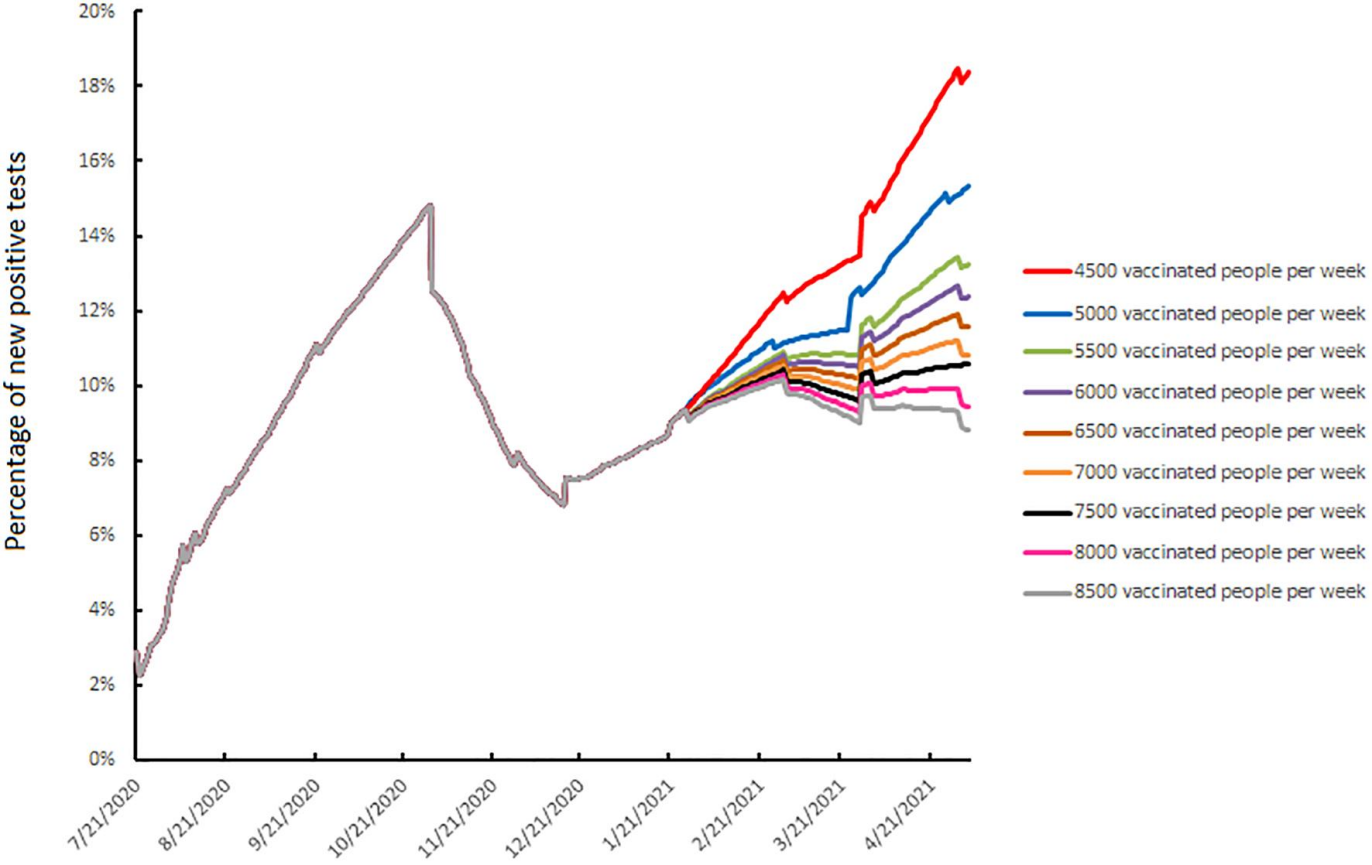
Daily spread of SARS‐CoV‐2 infection, 21 July 2020 to 5 May 2021 according to the vaccination strategy

The release measures taken during the last few months are likely to result in 7% of the urban Toulouse population being seropositive at the end of January, rising to 7.8% at the end of February and 8.8% at the end of March (Figure 2b). Vaccinating 4500 people each week from the beginning of January to the end of March (red curve, Figure 3) will result in seroprevalence increasing to 8.8% at the end of January, to 11.4% at the end of February and 14% at the end of March. SARS‐CoV‐2 spread will be slowed but not stopped. Increasing the number of people vaccinated from 4500 to 8500 by steps of 500 per week flattens the SARS‐CoV‐2 diffusion curve (Figure 3). Thus, if 7500 people are vaccinated each week, about 30,000/month, the SARS‐CoV‐2 seroprevalence would be 10% at the end of January, 13.8% at the end of February and 17.5% at the end of March (black curve, Figure 3). This vaccination strategy can control the spread of SARS‐CoV‐2, with the seropositive rate stabilized at 10% from the beginning of February to the end of May. Finally, increasing the number of vaccinations to 8500 per week, about 34,000/month, would result in the SARS‐CoV‐2 seroprevalence being 10.4% at the end of January, 14.6% at the end of February and 18.7% at the end of March (grey curve, Figure 3). The circulation of SARS‐CoV‐2 could be decreased using this strategy. Vaccination will undoubtedly continue beyond March 31, but this will have no influence on SARS‐CoV‐2 prevalence during the first 3 months of 2021.

### 3.3 Consequences of relaxing public health measures

As proactive vaccination strategies could keep the spread of SARS‐CoV‐2 under control, we assessed the date on which public health measures such as wearing masks could be relaxed without a resumption of the epidemic.

**FIGURE 4:**
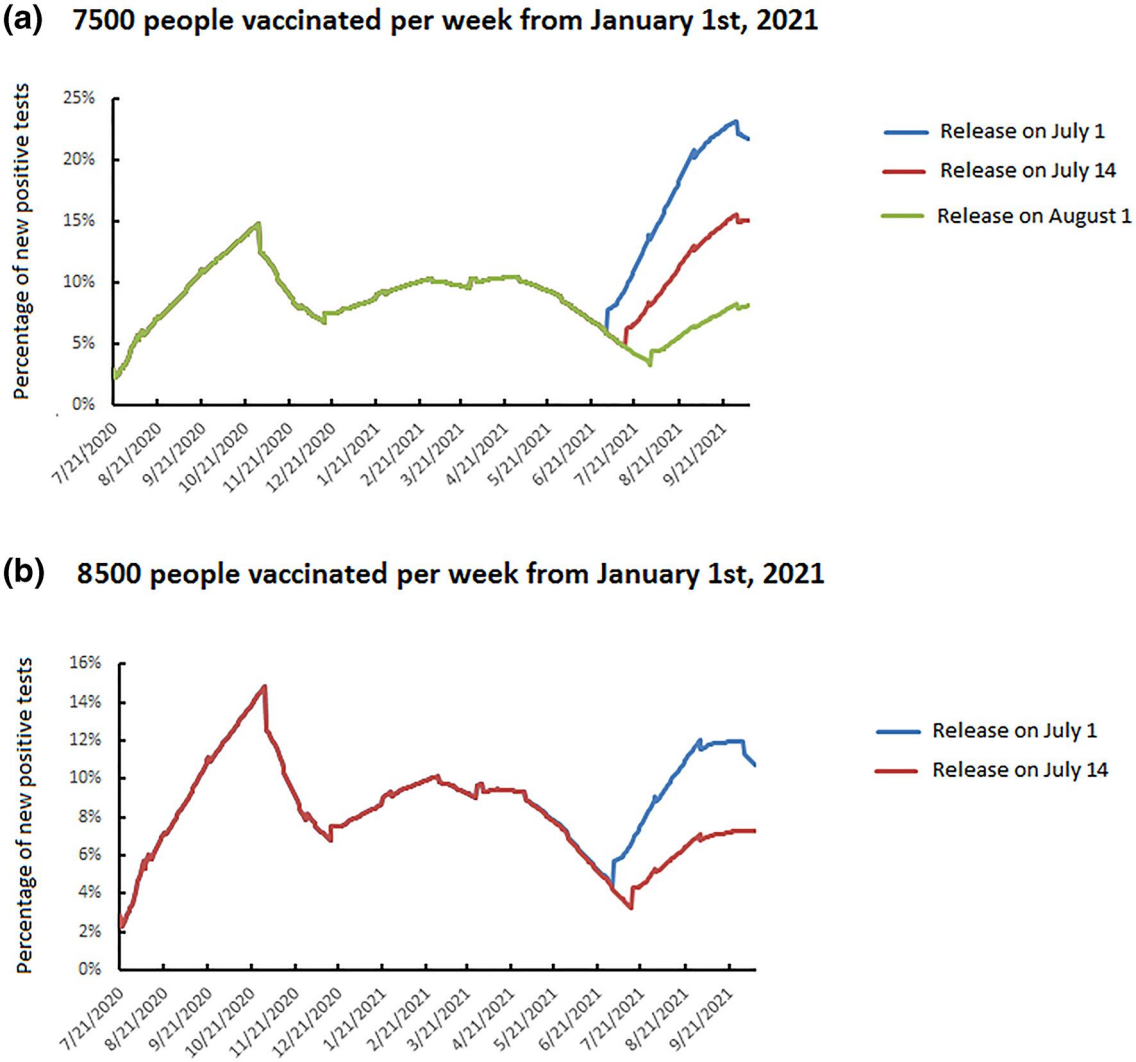
Daily spread of SARS‐CoV‐2 infection, 21 July 2020 to 9 October 2021 depending on the vaccine strategy and the release of public health measures: (a) 7500 people vaccinated per week from 1 January 2021; (b) 8500 people vaccinated per week from 1 January 2021

If an active vaccination campaign designed to control the spread of SARS‐CoV‐2 with 7500 people vaccinated per week (black curve, Figure 3) is adopted, it would be necessary to wait until 1 August 2021 before masks could be removed without a strong increase in the percentage of people testing positive (green curve, Figure 4a). If masks are removed on July 1 (blue curve, Figure 4a) or July 14 (red curve, Figure 4a), the rate of positive tests could become similar to those that led to the second lockdown on August 7 or early October. An even more massive vaccination campaign (grey curve, Figure 3), with 8500 people vaccinated per week from the beginning of January, would enable wearing masks to be stopped on July 14 (red curve, Figure 4b). Hence, immunity, owing to vaccination or infection, would be about 32% which is insufficient to achieve herd immunity. This indicates that strict hygiene rules and physical distancing will remain essential.

## 4 DISCUSSION

We have used a discretized SIR model to predict the dynamics of SARS‐CoV‐2 infections if various public health strategies are adopted. The second lockdown began on 29 October in France and has been gradually relaxed from 28 November 2020. We have shown how virus proliferation at the end of lockdown is a function of both the release date (and the level of circulation of the virus on that date) and the constraints retained following lockdown. Relaxing these constraints too much, for example by requiring only the wearing of masks, which corresponds to a 28% constraint on virus circulation,^3^, could allow the virus to rebound very quickly and overpower the hospitals.

Likewise, if lockdown is ended too soon, when the virus is still actively spreading, the epidemic could rebound even more rapid. For example, the first lockdown in France was ended when around 2% of the population tested positive. The strategy adopted by the French authorities restricts the spread of the virus, but would lead, without any further measure, to a percentage of positive tests similar to that which triggered the second lockdown in early March 2021.

However, an appropriate vaccination campaign, with 7500 people vaccinated per week throughout the first 3 months of 2021 (90,000 people over the 3 months), could keep the virus under control. The programme recommended by the French authorities requires the vaccination of primarily residents of nursing homes or long‐term‐care hospital units, the staff of these establishments over 65 or with comorbidities (from 1 January 2021), followed by people over 75 and healthcare workers over 50 or with comorbidities (from 18 January 2021), then people over 65 (expected as of mid‐February 2021).^18^ Based on the prevalence of SARS‐CoV‐2 in these age categories^19^ and the demography of the Toulouse urban area population,^20^ this will require vaccinating all 7500 residents of nursing homes or long‐term‐care hospital units and around 100 workers in these establishments with co‐morbidities or over the age of 65 (vaccination phase 1). The next group to be vaccinated would be the 57,500 people in urban Toulouse who are over 75 years old and the 5000 carers over 50 or with comorbidities (phase 2). Lastly, this would allow vaccination of 35.8% of over‐65‐year‐olds in urban Toulouse (phase 2). But even vaccinating 8500 people per week would result in only 43% of over‐65‐year‐olds living in urban Toulouse being vaccinated. A vaccination campaign that runs only for the first three months of 2021 is therefore insufficient to satisfy phases 1 and 2, since all people over 65 cannot be vaccinated. It might, however, be sufficient to control the spread of the virus.

We have assumed that the transmission of SARS‐CoV‐2 was identical regardless of the age of each individual or any individual characteristic. Although children could be less susceptible to SARS‐CoV‐2,^21^, ^22^ there is limited published evidence of age‐related differences in infectivity.^23^, ^24^, ^25^ Moreover, there may be errors in ascertaining the direction of transmission, leading to confusing differences in infectiousness with differences in susceptibility. We assumed that reinfection with SARS‐CoV‐2 after an initial infection or vaccination was unlikely. Existing neutralizing antibodies appear to have protected against reinfection people aboard a fishing vessel where there was a SARS‐CoV‐2 outbreak.^26^ Similarly, the passive transfer of antibodies can protect animals against COVID‐19^27^, ^28^ and the neutralizing antibody titre is correlated with protection after inoculation.^29^ Recent data indicate that an initial SARS‐CoV‐2 infection in a French population of healthcare workers protected 84.8% of them against reinfection for at least 167 days.^30^ This is consistent with a longitudinal study in the United Kingdom suggesting that previous infection resulting in antibodies to SARS‐CoV‐2 protects most people from reinfection for at least 6 months.^31^ We also assumed that the vaccinated people will not transmit the SARS‐CoV‐2 to healthy unimmunized people. This is only a hypothesis because phase 3 clinical trials of COVID‐19 vaccines were not designed to demonstrate prevention of transmission.^32^, ^33^, ^34^ However, there is a report that previous infection may not interrupt transmission of the Middle East respiratory syndrome coronavirus (MERS‐CoV) in camels.^35^ Likewise, inactivated polio vaccines protect against the disease but are less effective at reducing faecal excretion of the polio virus and, by extension, its transmission.^36^ Our model also assumes that the vaccine is effective whatever the subject's age, especially in the elderly. However, influenza vaccines are less effective in older people, in part because of immune senescence,^37^ which might also be true for COVID‐19 vaccines.

Our modelling indicates that public health measures like physical distancing, mask wearing, testing‐tracing and quarantine would still be needed for several months even with mass vaccination of people at risk. If these measures are not continued for the first 6 months of 2021, the seroprevalence of SARS‐CoV‐2 in the Toulouse population will be too low to prevent a rebound of the epidemic.^13^ Some simulation studies have shown that a new wave of virus replication can occur if less than 60% of a population is immunized.^13^, ^14^

Our study has several limitations. The forecasts obtained with this SIR‐type epidemiological model assume that its parameters remain stable over time. Like all mathematical models, there are potential biases associated with parameter estimation that can lead to biased projections. We have attempted to overcome this problem for the two parameters $\hat{c}$ and $\hat{q}$ that account for the impact of public health measures by correcting the predicted data using observed data.^3^ For other parameters like $R_{0}$, the herd immunity threshold is defined by 1 – 1/$R_{0}$, which implies that a higher $R_{0}$ requires a greater immune proportion of the population in order to block sustained transmission.^38^ The estimated virus proliferation rates resulting from the application of various public health measures also assume that the population must continue to adhere to these measures stably over time. For example, we assume that mandatory mask wearing would continue when the second lockdown is released just as it was at the end of the summer in the Toulouse urban area. And obviously, estimating the impact of vaccination on virus spread assumes that the sociological constraints and the vaccination campaign are widely adhered to, especially among priority populations.

Thus, the decisions of the French authorities concerning the end of lockdown could lead to a rapid resumption of the spread of the virus, but a massive, early and effective vaccination campaign should keep the spread of SARS‐CoV‐2 under control while maintaining massive testing, contact tracing and isolation. This conclusion is all the more valid with the emergence of new variants such as the UK strain of SARS‐CoV‐2.^39^ Although only six cases (around 1% among SARS‐CoV‐2 infected individuals) were detected in the Toulouse urban area between 1 January 2021 and 21 January 2021, subsequent studies are scheduled to assess its spread and the consequences for the model parameters.

## CONFLICT OF INTEREST

The authors have no conflict of interest to declare.

## AUTHOR CONTRIBUTIONS

Chloé Dimeglio and Jean‐Michel Loubes contributed to the model design and methodology. Marcel Miedougé, Jean‐Michel Mansuy and Jacques Izopet contributed to data collection. Chloé Dimeglio, Marcel Miedougé and JI contributed to data interpretation. Chloé Dimeglio and Jacques Izopet wrote the first draft of the manuscript. All authors contributed to critical revision of the manuscript and gave final approval.
